# Application Of Stable Isotope Analysis To Study Temporal Changes In Foraging Ecology In A Highly Endangered Amphibian

**DOI:** 10.1371/journal.pone.0053041

**Published:** 2013-01-15

**Authors:** J. Hayley Gillespie

**Affiliations:** Section of Integrative Biology, University of Texas at Austin, Austin, Texas, United States of America; Monash University, Australia

## Abstract

**Background:**

Understanding dietary trends for endangered species may be essential to assessing the effects of ecological disturbances such as habitat modification, species introductions or global climate change. Documenting temporal variation in prey selection may also be crucial for understanding population dynamics. However, the rarity, secretive behaviours and obscure microhabitats of some endangered species can make direct foraging observations difficult or impossible. Furthermore, the lethality or invasiveness of some traditional methods of dietary analysis (e.g. gut contents analysis, gastric lavage) makes them inappropriate for such species. Stable isotope analysis facilitates non-lethal, indirect analysis of animal diet that has unrealized potential in the conservation of endangered organisms, particularly amphibians.

**Methodology/findings:**

I determined proportional contributions of aquatic macroinvertebrate prey to the diet of an endangered aquatic salamander *Eurycea sosorum* over a two-year period using stable isotope analysis of ^13/12^C and ^15/14^N and the Bayesian stable isotope mixing model SIAR. I calculated Strauss’ dietary electivity indices by comparing these proportions with changing relative abundance of potential prey species through time. Stable isotope analyses revealed that a previously unknown prey item (soft-bodied planarian flatworms in the genus *Dugesia*) made up the majority of *E. sosorum* diet. Results also demonstrate that *E. sosorum* is an opportunistic forager capable of diet switching to include a greater proportion of alternative prey when *Dugesia* populations decline. There is also evidence of intra-population dietary variation.

**Conclusions/significance:**

Effective application of stable isotope analysis can help circumvent two key limitations commonly experienced by researchers of endangered species: the inability to directly observe these species in nature and the invasiveness or lethality of traditional methods of dietary analysis. This study illustrates the feasibility of stable isotope analysis in identifying preferred prey species that can be used to guide conservation management of both wild and captive food sources for endangered species.

## Introduction

Understanding foraging strategies of consumers is essential to understanding their trophic relationships and ecological roles. It is particularly important to understand these relationships when the consumers under study are rare, threatened and/or endangered. In the face of worldwide amphibian decline, it is more important than ever to understand the ecological roles amphibians fill in order to identify possible causes of these declines and how ecosystems may be affected by their loss 1. Amphibian species can account for staggering amounts of biomass in many of the world’s ecosystems 2,3, yet for many of these species we still know very little about their trophic relationships that ultimately impact ecosystem structure and function 4,5. Understanding long-term dietary trends for these species may be essential to assessing the effects of ecological disturbance such as habitat modification, species introductions or global climate change. Knowledge of short-term variation in prey availability and consumer foraging strategy can be crucial for understanding population dynamics in poorly understood species. Foraging strategies employed by consumers can have both ecological and evolutionary consequences. For example, populations of specialists may experience stronger selection than opportunists and generalists if prey availability changes rapidly due to fluctuating environments or levels of competition.

When foraging studies involve declining or threatened species, traditional methods of studying diet and resource use can become difficult or even inappropriate. Direct foraging observations may be impossible in rare species that inhabit obscure microhabitats or exhibit secretive behaviors. Stomach contents analysis by dissection or gastric lavage is often inappropriate for threatened or endangered species because of the high risk of mortality and morbidity associated with these invasive sampling methods. Furthermore, these traditional methods only take a snapshot of recent diet and may not accurately reflect long-term dietary patterns. Stable isotope analysis is an increasingly common tool in animal ecology and has several distinct advantages over traditional methods of dietary analysis, which can make it very useful in studies of rare or threatened species. This technology uses ratios of heavy to light stable isotopes found naturally in minute tissue samples from consumers to make inferences about diet and other ecological interactions without the need for direct observation. Stable isotope analyses reveal diet over the time period tissues are built in consumer tissue, and can reflect long-term dietary preferences. These analyses also have the ability to detect ecological relationships that can be unobservable using traditional methods alone, such as identifying soft-bodied organisms in the diet 6.

While stable isotope methods are becoming increasingly applied to conservation problems 7, amphibians in particular remain significantly underrepresented in the stable isotope literature, even compared to other herpetofauna. The fact that amphibians are typically small size and lack non-lethally sampled external tissues such as hair, feathers, scutes and scales may be preventing widespread non-lethal application of these cutting-edge tools to amphibian conservation research. Several herpetologists however, are beginning to use this technology successfully to examine the role of amphibians in ecosystems 4,8, quantify the impact of invasive species on native amphibians 9,10, assess dietary composition for understudied and threatened species 11,12, track seasonal movements 13 and even study mutualisms between native amphibians and endangered plants 14.

In this study, I examine the foraging ecology of the permanently aquatic endangered Barton Springs Salamander *Eurycea sosorum* in a series of observations of naturally occurring carbon and nitrogen stable isotopes from *E. sosorum* and potential prey from May 2007-June 2009. I also quantify prey availability in *E. sosorum* habitat using benthic macroinvertebrate surveys and use this information to calculate dietary electivity indices for *E. sosorum* throughout the two-year study period.

### Foraging Strategies & Conservation

Throughout this paper, I use foraging strategy definitions *sensu* Singer 15. *Generalists* are consumers that feed on a wide range of prey items, typically with no ranked preferences for particular food items and will thus select prey in proportion to their availability in the environment 16. *Opportunists* may also feed on a wide range of prey, but can change their diet to either take advantage of temporary availability of profitable resources, or to cope with temporary loss of staple resources (this is sometimes referred to as *diet switching*) 17. *Specialists* feed on one or a few preferred prey items regardless of availability and do not exhibit diet switching. Strong specialization is usually inferred when a predator selects one or more prey disproportionately to its availability in the environment. I use descriptions of foraging ecology *sensu* Singer 15 and Ivlev 18, who define *preference* to describe a behavioral characteristic of the predator; *acceptability* as a property of the prey item and *electivity* as property of the predator-prey interaction (described by the proportion of prey items in the diet relative to their availability in the environment).

Knowledge of foraging strategies of rare and endangered species can contribute to their conservation in several ways. First, identifying the most important prey species allows habitat managers to focus efforts on maintaining self-sustaining populations of essential prey. Second, identifying foraging strategies and essential prey allows managers to anticipate how target species may react to changes in prey populations. For example, if endangered species are strict specialists, habitat managers must insure that preferred prey populations are sustained or are cultured in captivity. For species capable of diet switching, maintaining habitat that supports a diverse prey community may be more appropriate. Finally, captive breeding programs are often limited by information about the diet of target species, and the ability to culture wild prey in captivity. Identifying key prey species allows for captive propagation efforts to become more focused and effective.

In previous publications, *Eurycea sosorum* has been assumed to be a generalist predator that relies heavily on the amphipod *Hyallela azteca* Saussure for food because *H. azteca* is very common in *E. sosorum* habitat 19,20,21. If *E. sosorum* is indeed a generalist predator, I expect stable isotope analysis to show that adult *E. sosorum* feeds on different prey species in proportion to their availability in Eliza Spring, and that there should be little variation in diet among individuals sampled at the same time.

## Materials And Methods

Work was conducted in Eliza Spring (Travis County, Texas, USA), which currently hosts the largest population of the Barton Springs Salamander *Eurycea sosorum*. Total *E. sosorum* density in Eliza Spring ranged from 1.29 to 16.60 individuals/m^2^ (88 to 1,210 total individuals) during this study (City of Austin, unpublished data). *Eurycea sosorum* is a permanently aquatic stream salamander that is known from only four freshwater springs collectively known as Barton Springs in the city of Austin, Texas, USA that are distributed over a total land area of approximately 0.05 km^2^ 20,22,23. Since the late 1800s, three of the four springs have experienced habitat modifications including dams and changes in riparian vegetation that have altered spring hydrology and terrestrial-aquatic habitat linkages 21,24. Salamanders inhabit benthic surfaces near spring outflows, and hide underneath rocky substrate that is free from sediments; they are also capable of living in subterranean habitat within the Edwards Aquifer 20. Thus, it is extremely difficult to directly observe foraging behavior in this species.

**Figure 1:**
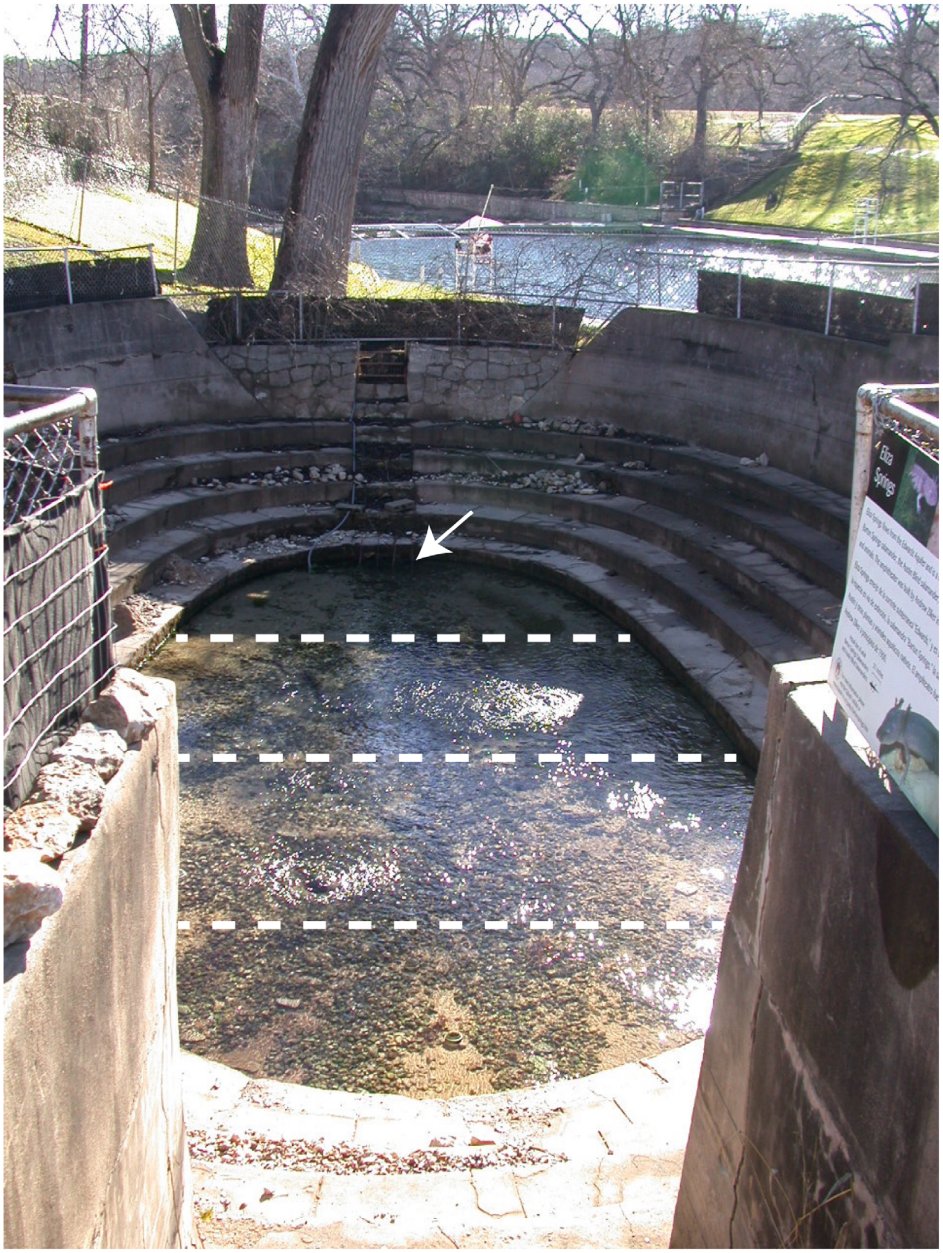
Eliza Spring, Zilker Park, Austin (Travis County), Texas and Transect Lines for Invertebrate Sampling. The lower dam of Barton Springs Pool, which hosts another population of *Eurycea sosorum*, is visible in the distance. Arrow marks pipe where water exits Eliza Spring. Dashed lines indicate transects along which macroinvertebrate samples were taken.

Because Eliza Spring is now surrounded by a concrete structure (Figure 1), there is little remaining riparian vegetation. A few clumps of aquatic macrophytes are found within the spring, including *Ludwigia repens* Forster, *Bacopa monnieri* Pennell and *Eleocharis* sp. The aquatic moss *Amblystegium riparum* Hedwig grows on the vertical concrete surfaces, periphyton covers benthic surfaces and several species of filamentous algae grow on benthic surfaces to different degrees throughout the year. The invertebrate community in Eliza Spring is typical of high-order spring-fed streams, with high endemism, relatively low diversity and species characteristic of cool, fast-flowing shallow waters 21,25,26 including the amphipod *H. azteca*, water penny larvae *Psephenus texanus* Brown & Arrington, midge fly larvae (family Chironomidae), caddisfly larvae (*Helicopsyche* sp.), heptageneiid and baetid mayfly larvae, planarian flatworms (*Dugesia* sp.), odonate larvae, small annelid worms and ostracods.

### Macroinvertebrate Abundance Sampling

I measured benthic macroinvertebrate densities at Eliza Spring approximately monthly from November 2007 through June 2009 (N = 16 sampling dates) using a stratified random sampling method along a flow gradient using Hester-Dendy artificial substrate samplers 27 and a Hess-type benthic sampler equipped with a 363µm mesh net 28. Appendix S1 provides a detailed description of the sampling protocol. I calculated average density of macroinvertebrates per m^2^ on each date from both Hester-Dendy samplers (N = 6 per date) and Hess samplers (N = 3 per date; total of N = 9 samplers per date). I calculated relative abundance of potential prey species on each macroinvertebrate sampling date by dividing the number of individuals of a single species of invertebrate divided by the number of individuals of all species on each date. Because some invertebrate species were too large or clung too tightly to benthic substrate for salamanders to feasibly consume, I narrowed the set of prey items included in stable isotope analyses using the selection criteria presented in Appendix S1. The prey items that met these criteria and were included in stable isotope analyses were the amphipods *Hyalella azteca* (hereafter “amphipods”), *Dugesia* sp. planarian flatworms (hereafter “planarians”) and midge fly larvae in the family Chironomidae (hereafter “chironomids”).

### Stable Isotope Analyses

For all isotope sample collection, I froze tissue samples (tail clips for salamanders, whole prey) in the field on dry ice. Tail clipping methods are presented in Appendix S1. I dried samples at 50°C for seven days, ground each sample into a homogenous powder with a mortar and pestle and weighed 1 mg into 3×5 mm tin capsules (Costech Analytical Technologies, Valencia, California, USA). Samples were analyzed at the University of California at Davis Stable Isotope Facility for natural abundance ^13/12^C and ^15/14^N isotope ratios on a Europa Hydra 20/20 continuous-flow IRMS mass spectrometer equipped with an elemental analyzer. Resultant values are reported as the difference between stable isotope ratios of tissue samples and international lab standards (Vienna Peedee Belemnite for ^13/12^C; atmospheric N_2_ for ^15/14^N), and are referred to as delta (δ) values in units of per mil (‰) 29. By comparing δ values for one or more isotopes in the food item(s) with δ values in consumer tissue, one can determine the relative contribution of each isotopically distinct food item to a consumer’s diet 30.

Proportional contributions of potential prey species to the diet of adult *E. sosorum* were estimated using the Bayesian stable isotope mixing model Stable Isotope Analysis in R (SIAR), which incorporates both variation in stable isotope values from both consumers and prey and variation in isotopic discrimination factors 31. Isotopic discrimination of δ^13^C‰ is typically small (≈1‰) making it an indicator of diet carbon source, whereas δ^15^N‰ typically enriches 3–4‰ in consumers compared to their prey, making it an indicator of relative trophic level 32. I used isotopic discrimination estimates of 2.31±0.22‰ for δ^15^N‰ and no significant δ^13^C‰ discrimination from a study in which I compared isotope values from captive-born *E. sosorum* with isotope values of the commercial bloodworm diet that they had been fed since hatching (J.H.G. unpublished data). I used default SIAR modeling specifications with 200,000 iterations and the estimated proportional contributions of each source item to consumer diet are Bayesian posterior distributions (and associated residual errors) and are graphically summarized by SIAR using 95%, 75% and 25% Bayesian credible intervals for each prey type for each sampling date 31.

I ran one mixing model for each sampling date. Data input into each model included the isotopic discrimination factors above, δ^13^C‰ and δ^15^N‰ values from each individual *E. sosorum* on a sampling date and the study-wide mean ±1 SD of δ^13^C‰ and δ^15^N‰ for each prey species (Table 1). I used study-wide means of prey isotope values rather than individual sampling date means because the portion of the tail clips that contains bone could potentially integrate diet for up to two years (J.H.G. unpublished data). I ran one mixing model per sampling date because the skin and muscle portions of the salamander tail clips used in this study may turn over more quickly than the bone portion (on the order of weeks or months). To illustrate the degree of intrapopulation dietary variation over the course of the study, I present stable isotope values from individual salamanders.

**Table 1:** Study-wide means ±1SD of stable isotope values and percent carbon and nitrogen for *Eurycea sosorum* and prey tissues.

	δ^13^C‰	δ^15^N‰	% C	% N	Joules/mg*	% Protein*
	−33.34±1.78	9.41±0.70	40.12±0.10	8.74±1.50		
*Eurycea sosorum*	(−38.23, −28.54)	(7.50, 11.68)			–	–
	N = 160	N = 160	N = 80	N = 160		
	−32.53±1.41	4.72±0.68	33.75±3.80	7.15±1.28	16.07±3.95	33.10±10.61
Amphipod	(−36.23, −29.04)	(2.51, 6.35)				
	N = 132	N = 132	N = 62	N = 62	N = 85	N = 25
	−34.53±1.14	8.61±0.75	51.28±7.15	9.96±2.10	25.62±0.40	66.10±3.09
Planarian	(−38.03, −31.22)	(7.08, 10.80)				
	N = 78	N = 78	N = 20	N = 20	N = 4	N = 2
	−35.02±1.61	6.15±0.68	42.35±8.26	7.70±1.71	21.11±3.62	
Chironomid	(−39.44, −32.04)	(4.37, 7.17)				–
	N = 31	N = 31	N = 8	N = 8	(N = 15)	

Range of isotope values are presented in parentheses.

* Indicates data from the Brey *et al.* (2010) 36 global database of body composition of aquatic organisms. Dashes indicate no data was available.

doi:10.1371/journal.pone.0053041.t001

### Foraging Strategy Of *Eurycea Sosorum*

To determine whether *E. sosorum* forages in proportion to prey availability (a generalist feeding strategy), I calculated Strauss’ 33 linear index of electivity for each stable isotope sampling date (except May 2007, for which there was no previous macroinvertebrate abundance data). The Strauss index is calculated using the formula *L_0_* = *r_i_ – p_i_* where *r_i_* represents the relative proportion of item *i* in the diet and *p_i_* represents the relative abundance of item *i* in the environment. Values of the Strauss index range from +1 (strong electivity) to −1 (low electivity or avoidance), with values near zero indicating no electivity or foraging in proportion to availability. I used mean SIAR mixing-model-estimated proportions of each potential prey species in the diet of *E. sosorum* for *r_i_*. For *p_i_*, I calculated the average relative abundance of each potential prey species since the previous isotope sampling date.

## Results

### Macroinvertebrate Abundance

**Figure 2:**
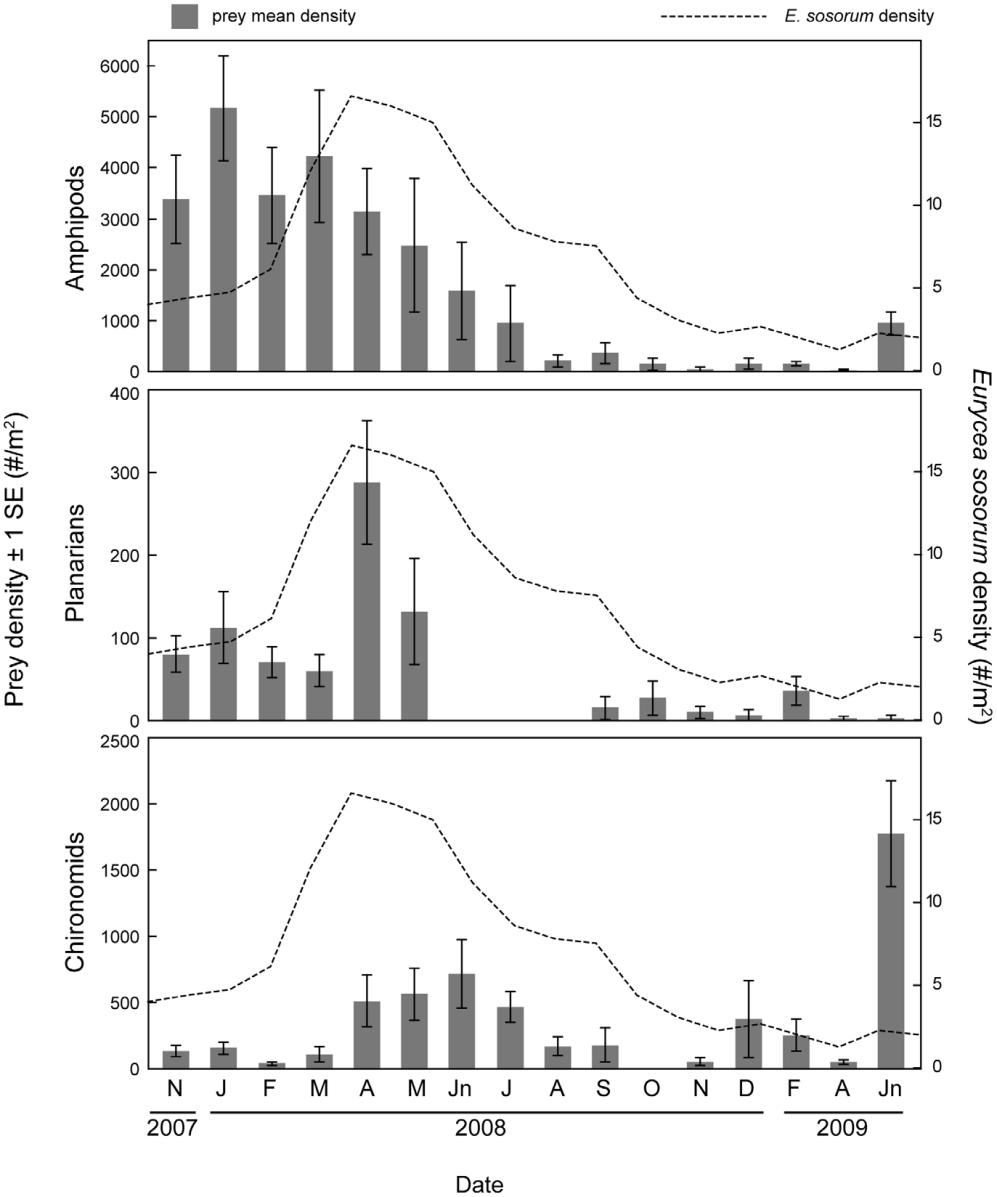
Mean ±1SE prey density and *Eurycea sosorum* density from Eliza Spring. N = 9 invertebrate samples per sampling date. Higher SE reflects more patchy spatial distribution of prey between sampling points. Only results for the three invertebrate species that met the criteria outlined in Methods are shown.

*Eurycea sosorum* population density peaked between April and June 2008, after which invertebrate populations experienced a subsequent sequence of declines (Figure 2). Planarian density declined to near zero within two months of the April 2008 peak in *E. sosorum* density, followed by a similar decline in chironomid density in October and November 2008. The amphipod population, however, experienced a more gradual decline (Figure 2). Coincident with the decline of the planarian population, a regional emergence event of chironomid larvae was observed during summer 2008 (J.H.G., personal observation) and chironomid density subsequently declined to near zero in October and November 2008.

### Stable Isotope Analyses

**Figure 3:**
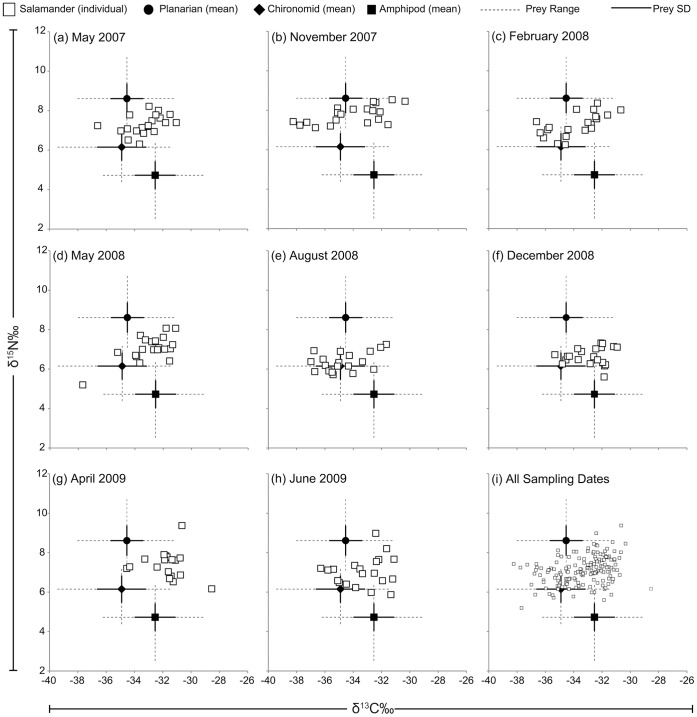
Stable isotope biplots of individual *Eurycea sosorum* and mean prey items. 2.31‰ is subtracted from each salamander δ^15^N‰ value to reflect isotopic discrimination. Prey stable isotope values are shown as study-wide means ±1SD.

**Figure 4:**
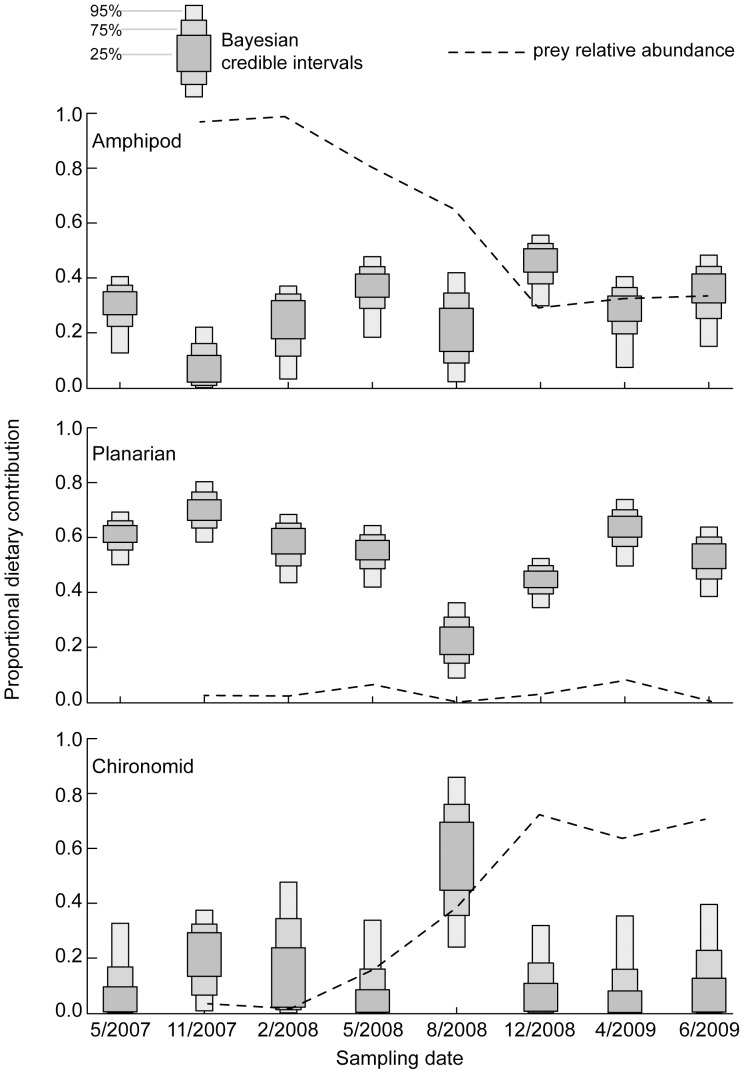
Mixing model estimated contributions of amphipods, planarians and chironomids to the diet of *Eurycea sosorum*. Bayesian credible intervals show estimated contributions of each prey item to the diet of *E. sosorum* derived from the stable isotope mixing model SIAR. Relative abundance of prey uses same scale on Y-axis.

**Figure 5:**
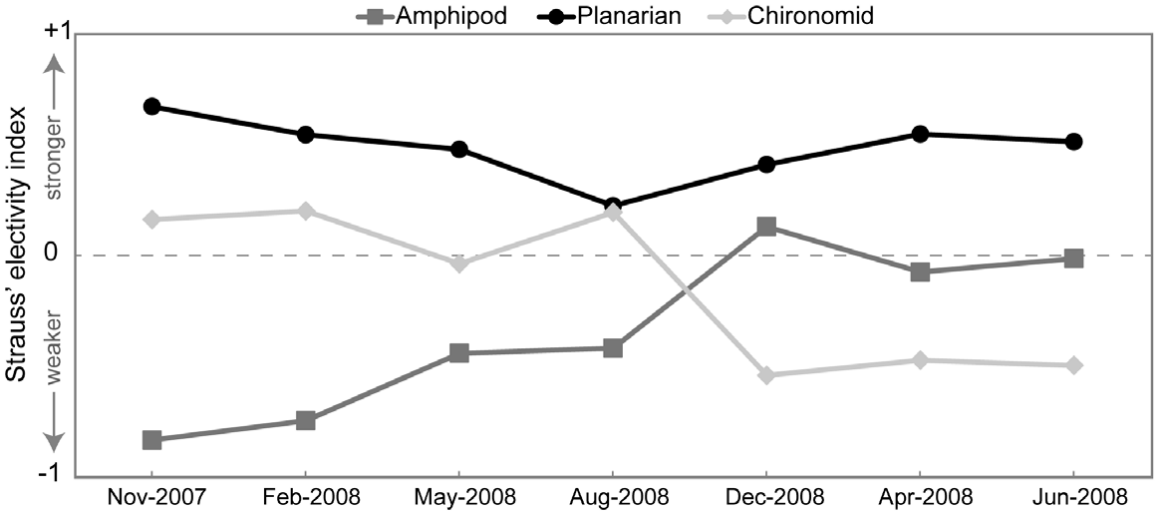
Strauss’ linear electivity index for amphipods, planarians and chironomids in the diet of adult *Eurycea sosorum*. Values near +1 indicate high selection despite low availability in the environment (strong electivity), values near zero indicate foraging in proportion to availability in the environment (no electivity), and values near −1 indicate that prey is not selected despite high availability in the environment (avoidance).

Stable isotope values of prey and salamanders were consistent with expectations that δ^15^N‰ enriches with increasing trophic level (Table 1; 32). A large amount of variation in δ^13^C‰ was observed between individual salamanders both within single sampling dates as well as over the course of the study (Figure 3), with November 2007 having the greatest range in δ^13^C‰ between individuals (7.9‰; Figure 3b). Statistical comparisons between prey contributions estimated by SIAR and prey densities are hampered by the nature of the mixing model output, which is simple proportions (not frequencies). However, visual comparisons of estimated *E. sosorum* diet and relative densities of prey items (Figure 4), as well as Strauss electivity indices (Figure 5) clearly indicate that salamanders selected prey out of proportion to availability in the environment. In addition, diet switching may be indicated by the shifts in salamander diet observed in August 2008 (Figure 4). Mixing model estimates of amphipod contribution to the diet of *E. sosorum* are far below proportional availability until December 2008, when availability and average mixing model estimates are similar (Figure 4). Though amphipods had the highest relative abundance for most of the study, mixing model results indicate that they contributed a maximum of 56% to *E. sosorum* diet in December 2008. Strauss electivity indices for amphipods were negative for most of the study, also indicating low electivity (Figure 5).

Planarians are available at much lower relative abundance than mixing model estimates of diet (Figure 4), and Strauss electivity indices indicate a high degree of electivity for planarians (Figure 5). Selection of planarians is lowest in August 2008 (8%–36%; mean 22%) following near depletion of the planarian population from the environment, to which *E. sosorum* apparently responded by increasing consumption of chironomids and amphipods in December 2008.

Though chironomid relative abundance increased over the course of the study, their contribution to *E. sosorum* diet was low in the last few sampling dates (Figure 4). Strauss electivity indices for chironomids were also lowest during the last three sampling dates (Figure 5). For all but one sampling date (August 2008), the distributions of mixing model solutions for chironomids included 0% and were heavily skewed towards low values (Figure 5). Highest contribution of chironomid larvae was August 2008 (24–85%; mean 55%), which followed a decline in both amphipods and planarian densities and was associated with a regional emergence event for chironomids in summer 2008.

## Discussion

The use of stable isotope analysis in this study revealed several important aspects of the foraging ecology of *Eurycea sosorum* that were previously unknown. First, the timing of prey population declines (Figure 2), stable isotope mixing model results (Figure 4) and Strauss electivity indices (Figure 5) during the course of this study all demonstrate that planarians – not amphipods – are the primary food source for adult *E. sosorum* at the population level. This result highlights a previously unrecognized food source for this highly endangered amphibian. Because amphipods are abundant in many springs where neotenic *Eurycea* occur, they have been assumed to be the main prey for this group of salamanders 19,20,21. However, results here indicate that amphipods only become part of the salamander's diet when other prey species (planarians and chironomids) become rare. If *E. sosorum* were a generalist predator, amphipods (the most abundant macroinvertebrate during most of the study) should have been the most consumed prey item on all sampling dates. Though stable isotope analyses indicate that amphipods do contribute to the diet (the highest estimate is 11–61% in December 2008), electivity indices show that amphipods were avoided during the first half of this study (Figure 5).

Results here also demonstrate that diet composition among individual salamanders is more variable than previously thought. If individual *E. sosorum* were all feeding in a similar way, one would expect much less variation in δ^13^C‰ among individual salamanders (indicating all individuals consumed similar proportions of prey). Instead, δ^13^C‰ varied greatly among individuals on some sampling dates (Figure 3). This intraspecific variation does not seem to be due to morphometric differences between individuals, as all salamanders sampled for this study were of the adult size class and extremely similar in size (mean snout-vent length ± SE = 30.45±0.53 mm, N = 120). Extreme δ^13^C‰ values for some individual *E. sosorum* indicate that these individuals may have been eating a very different combination of prey than much of the population (i.e. Figure 3b shows individuals with extremely low δ^13^C‰, Figure 3g shows some extremely high δ^13^C‰). Chironomid larvae are also known to feed on biofilms containing methane-oxidizing bacteria, which is typically depleted in δ13C‰ 34. Although often associated with anoxic conditions in lakes and ponds 35, this phenomenon was recently documented in headwater streams 36. Depleted δ13C‰ values observed in this study for some chironomid larvae (minimum −39.43‰), along with mixing model results for August 2008 could be further evidence that biogenic methane ultimately contributes to top consumers in freshwater systems.

This study also provides evidence of temporary diet switching from a planarian-dominated to chironomid-dominated diet in August 2008. Diet switching indicates an opportunist foraging strategy in which a predator may change its diet to take advantage of temporarily available or more acceptable prey, or to replace a preferred species that has become temporarily unavailable with a lower ranked food source. In this study, both patterns emerged. The increased proportion of chironomids in the diet of *E. sosorum* in August 2008 may have occurred because of a combination of increased availability of chironomids in benthic habitat as these insects emerged from their cases coupled with coincident declines in planarian density. Regardless of the specific mechanism, these results clearly demonstrate that *E. sosorum* are opportunists, with the capability to diet switch as prey communities fluctuate.

Planarians have several obvious advantages as prey for *E. sosorum*. Planarians are slow-moving, prefer microhabitats with relatively low-flow conditions 37 and lack a hard exoskeleton, all of which may reduce handling time for these prey items. Percent carbon and nitrogen content in prey tissues can be used as proxies for relative energetic and protein content, respectively (as in 38). Planarian tissue had the highest percent carbon and nitrogen of the three potential prey in this study; amphipods had the lowest (Table 1). These findings are consistent with data from Brey *et al.*’s 39 global database of body composition of aquatic organisms, which also shows that planarians have higher energy and protein content per unit dry mass than amphipods and chironomids (Table 1).

It is not surprising that published accounts of wild salamanders feeding on soft-bodied planarians are scant given that most studies of salamander diet are based on stomach and fecal contents analysis (but see 40,41). Interestingly, larval *Salamandra salamandra* and *Salamandrina perspicillata* have been observed feeding on planarians (*Dendrocoelum* sp. and *Dugesia* sp.) in spring-fed cave streams in Italy (Raoul Manenti, personal communication). Thus, planarians may be an important but overlooked prey item for aquatic salamander species.

### Study Limitations

Any stable isotope analysis of diet is constrained by the availability of potential prey at the time of sampling. In this study, the three-year absence of baetid mayfly larvae from Eliza Spring prevented me from incorporating this prey item into isotope analyses. However, the extremely long duration of their absence suggests that baetid mayfly larvae are probably not essential prey for *E. sosorum*. In addition, uncertainty in turnover times of consumer tissues can make it difficult to choose an appropriate time span over which to incorporate prey isotope values into mixing models. To make sure my choice of study-wide means for prey isotope values in mixing model analyses did not affect overall conclusions, I compared mixing model results using several variants of lagged prey isotope values from individual sampling dates (e.g. current sampling date only, previous sampling date only, mean of current plus one previous sampling date and mean of past two sampling dates) and all model variants had very similar results to those presented herein (J.H.G. unpublished data). Thus, uncertainty regarding exact salamander tail clip turnover time does not change my conclusions about *E. sosorum* diet in this study.

### Conservation Implications

In this study, stable isotope analysis revealed novel foraging behaviors that have direct relevance to habitat management and restoration in the field as well as diet selection for captive breeding facilities. For example, the observation that *E. sosorum* may be capable of diet switching means that temporary declines in preferred prey density may not pose a significant threat to *E. sosorum* populations, but the long-term effects of such depletions are as yet unknown. In Eliza Spring, habitat management and restoration activities could seek to promote flow heterogeneity in order to promote growth of *Dugesia* populations 37. Rearing planarians in captivity could be used to supplement the diets of captive *E. sosorum* using methods developed by Kolasa & Tyler 42). Additionally, because this study indicated that some individual *E. sosorum* may have different dietary preferences than the average of the population it suggests that feeding a combination of prey items to captive *E. sosorum* could improve captive breeding success.

### Amphibians & Stable Isotope Analyses

In conclusion, stable isotope analysis has the potential to revolutionize the study of amphibians and contribute greatly to their conservation. However, there is still much work to do before use of stable isotope techniques can become commonplace for amphibians. First, there is a need for more laboratory studies that examine turnover time and isotopic discrimination in amphibian tissues, because some metabolic processes that influence assimilation of stable isotopes in reptiles and amphibians may be quite different from those of the well-studied endotherms 43,44,45. Second, there is a need for improved non-lethal and non-invasive techniques for sampling isotope ratios from amphibians, especially endangered species. Recently developed methods to sample the mucous coat of earthworms 46 and fishes 47 could greatly increase applicability of stable isotope analyses if adapted for use on amphibians. Such a technique would negate the need for toe or tail clipping, allow repeated measures on individual animals and provide a tissue with rapid turnover time. In addition, examination of fecal pellets (either by dissection and identification of invertebrate remains or stable isotope analysis of the pellet itself) could be a useful complement to stable isotope analyses of body tissue. Gastric lavage has been used in some larger salamander species 48, but Diaz 49 reports that attempted gastric lavage on *Eurycea nana* resulted in injury or death without effectively flushing stomach contents. Interestingly, the observation that two *E. sosorum* individuals in this study regurgitated the stomach contents when their gills contacted Bactine ® anesthetic warrants exploration as a non-invasive method of gastric lavage in small salamanders. As amphibians are quickly becoming some of the most threatened taxa on Earth, it will be especially important to rapidly develop these improved isotopic methods and apply them in systems where the ecological roles of amphibians may be greatly underestimated or are as yet unknown 3,8.

## Supporting Information

Appendix S1 **Additional methodological details that includes, Freshwater Macroinvertebrate Sampling Protocol, Selection Criteria for Potential Prey Species in Stable Isotope Analysis, and Collection of Eurycea sosorum Tail Tissue and Invertebrate Tissue for Stable Isotope Analysis.**
(DOC)
