# Supplementary material for: Application Of Stable Isotope Analysis To Study Temporal Changes In Foraging Ecology In A Highly Endangered Amphibian
Source: PLoS One. 2013 Jan 15;8(1):e53041. doi: 10.1371/journal.pone.0053041 (PMC3546114; doi:10.1371/journal.pone.0053041)
Supplement: Appendix S1 — (DOC) [file pone.0053041.s001.doc]

*Supporting Information For:* Application of stable isotope analysis to study temporal changes in foraging ecology in a highly endangered amphibian.

## Additional Methods

*Freshwater Macroinvertebrate Sampling Protocol*

Three sampling transects were established at equally spaced intervals across Eliza Spring’s longest axis. Two HD samplers (surface area 0.07 m^2^ each) were deployed at random distances along each transect line (distances chosen by random number generator). Prior to the first sampling date (November 2007), HD tiles were allowed to colonise in the springs for 30 days, wiped clean with a soft brush and then re-deployed for a further 30 days. On each sampling date, HD samplers were gently collected in a fine-mesh net (to avoid loss of macroinvertebrates as they were lifted out of the water), placed in a bucket and scrubbed gently with a soft brush. Invertebrates were preserved in 70% ethanol in the field. Preserved samples were sorted by taxon and counted in the laboratory. After processing HD samplers, a third randomly-selected distance along each transect determined the sample location for the Hess-type sampler (which covered 0.03 m^2^ benthic surface area). Because monthly salamander surveys at Eliza Spring disturb benthic substrate, invertebrate collections were conducted one to three days prior to each survey; HD samplers were then re-deployed at new randomly-chosen distances along each transect after each salamander survey. The sample interval was approximately 30 days. Though there were slight differences in the estimated total density of amphipods and planarians between HD and Hess samplers, the patterns of population trends of amphipods, planarians and chironomid larvae were strikingly similar between the two methods.

### Selection Criteria for Potential Prey Species in Stable Isotope Analysis

### I used two criteria to determine which macroinvertebrate species to include in stable isotope analyses by assessing which species could be feasible prey for E. sosorum. First, because most salamanders are gape-limited predators, I included any prey smaller than 10 mm in any dimension (the largest observed head length of E. sosorum in this study). Second, because E. sosorum feeds by suction, I included prey that could be removed from benthic substrate using suction from a small pipette. Some macroinvertebrates that fit both of these criteria (e.g. mayfly larvae, ostracods, annelid worms) were so rare during the course of this study that despite intense sampling effort over several days, I could not collect enough of these species for stable isotope analysis. Baetid mayfly larvae, for example, were present during preliminary visits in November 2006, but were completely absent from 2007 to 2009. Using these criteria, the final prey items included in stable isotope analyses were the amphipods Hyalella azteca, Dugesia sp. planarian flatworms and midge fly larvae in the family Chironomidae.

### Collection of Eurycea sosorum Tail Tissue and Invertebrate Tissue for Stable Isotope Analysis

In order to minimize disturbance of the wild population, collection of isotope samples from *E. sosorum* were taken during monthly population surveys conducted by City of Austin biologists. Because *E. sosorum* is an endangered species, a non-lethal tissue sample large enough for stable isotope analysis had to be identified. Tail clips were chosen because individual *E. sosorum* are too small and fragile to collect blood or other tissues. Loosing part of the tail tip is part of salamander natural history, and tail tips regenerate within several weeks (JHG personal observation), thus posing no long-term harm to the animals. Tail clips contain skin, muscle and bone, and thus represent a mixture of both rapidly and slowly generated tissue, rather than just one tissue type. The advantage of this is that tail clips represent long-term dietary patterns, rather than a snapshot of salamander diet. The disadvantage is that the time scale over which tail clips integrate diet is more difficult to identify, with skin and muscle fractions able to turn over relatively quickly, and the bone fraction turning over more slowly. In a related set of diet-switching observations on *E. sosorum* in which I reared wild-caught salamanders on an isotopically distinct diet, I observed approximately 2‰ and 10‰ changes in tail tissue δ^15^N‰ and δ^13^C‰, respectively, within 266 days (J.H.G., unpublished data). Complete equilibration of tail clips with the isotopically distinct diet, however, could take up to two years (JHG unpublished data). Micro-dissection of tail clips was attempted, but it was not feasible to cleanly separate bone from muscle and skin in 5mm tail clips. In a study of the diet of an endangered frog, Najera-Hillman et al. [1], observed that δ^13^C‰ and δ^15^N‰ of frog toe clips (like salamander tail clips, these contain a mixture of skin, muscle and bone) did not significantly differ from isotope values of muscle only.

Tail clipping procedure occurred in the field, and took less than five minutes per animal. Five individual salamanders were caught using mesh nets from each of four sampling quadrants of Eliza Spring (approximately 20 salamanders per sampling date, N=160 total). Salamanders used in this study were all adults (mean snout-vent length ± SE = 30.45 ± 0.53 mm) and only salamanders that had undamaged, normally-developed tails were used. When caught, each salamander was placed in an individual plastic container filled with spring water that was floated in Eliza Spring to keep them cool. For clipping, each animal was transferred from the container to a shallow glass dish with fresh spring water by lifting it gently onto a moist piece of fine mesh. While briefly out of the water on the mesh, I applied one to two drops of the topical anesthetic and antiseptic Bactine ® (Bayer Corporation; Benzalkonium CL 0.13%, lidocaine HCL 2.5%) to the tip of the tail with a 5-ml plastic pipette [2]. Care was taken to prevent any other part of the salamander’s body from contacting the anesthetic, but on two occasions the salamander turned 180˚ after application of the anesthetic and touched its head and gills to the tail. Interestingly, this caused both salamanders to immediately regurgitate the stomach contents. Both salamanders became fully anesthetised with an effect very similar to that of MS-222 (JHG personal observations) and regained full ambulatory function approximately 10 minutes after being transferred to fresh water. Total length, snout-vent length, tail length and head width measurements (mm) and identifying photographs were taken for each salamander. Iris scissors (BioQuip Products, Rancho Dominguez, CA) were used to take a 5-mm clip from the distal tip of the tail. Clips were rinsed immediately with clean filtered water, stored in ½ dram glass vials sealed with Parafilm (Pechiney Plastic Packaging Company, Chicago, IL) and frozen on dry ice. After clipping, salamanders were returned to individual plastic containers with fresh spring water and monitored for at least two hours before being released back into Eliza Spring at the conclusion of each salamander survey. No complications or mortality from tail clipping were observed in this study during the clipping procedure or monitoring period. Clipping equipment was cleaned with rubbing alcohol between each clip. Tail clipping was conducted under University of Texas IACUC protocol #07092602 and under the auspices of federal (US Fish & Wildlife Permit TE-83851) and state (Texas Parks & Wildlife Permit SPR-1005-1515) permits held by the City of Austin under the supervision of L. Dries.

Invertebrates were collected whole, rinsed with filtered water, stored in ½ dram glass vials sealed with parafilm and frozen on dry ice in the field. Because of the small size of some invertebrates, I used composite samples of 20 planarians, five amphipods and two chironomid larvae to reach 1 mg of dry weight for stable isotope analysis. Planarians dried to a film which was scraped out of glass vials with a small metal weighing scoop to be used for isotope analysis.

## Literature Cited

1. Najera-Hillman E, Alfaro AC, Breen BB, O’Shea S (2009) Characterisation (δ^13^C‰ and δ^15^N‰ isotopes) of the food webs in a New Zealand stream in the Waitakere Ranges, with emphasis on the trophic level of the endemic frog *Leiopelma hochstetteri*. New Zealand Journal of Zoology 36: 165-176.

2. Green DE (2001) *Restraint and handling of live amphibians.* Amphibian Research and Monitoring Initiative Standard Operating Procedure No. 100. ACUC tracking no. 2001-005. Madison: USGS National Wildlife Heath Center.
